# Methane emissions from fresh dairy cattle and pig slurry

**DOI:** 10.1002/jeq2.70186

**Published:** 2026-04-29

**Authors:** E. G. G. van Boxmeer, H. J. Smit, N. Verdoes

**Affiliations:** ^1^ Wageningen Livestock Research Wageningen University & Research Wageningen The Netherlands

## Abstract

During manure storage, methane (CH_4_) is produced by anaerobic decomposition of organic matter by methanogens. Frequent removal and further processing of manure from the barn can reduce CH_4_ emissions. However, little is known about how much CH_4_ is lost during the adaptation of methanogens to the changing environment from the gut to storage. The objective was to determine the breakdown of organic matter and emission of CH_4_ from dairy cattle and pig slurry in the first 3 days after excretion. CH_4_ and carbon dioxide (CO_2_) emissions from fresh slurry (<1 h old) were measured in climate respiration chambers. Three treatments were studied: (1) dairy cattle slurry with a temperature of 15°C (CS15), (2) dairy cattle slurry with a temperature of 20°C (CS20) and (3) pig slurry with a temperature of 20°C (PS20). CH_4_ emissions from both dairy cattle and pig slurry were minimal during the first 3 days after excretion. Temperature influenced emission rates, resulting in higher CH_4_ and CO_2_ emissions from CS20 than from CS15. Cumulative CH_4_ and CO_2_ emissions from PS20 were not significantly different from those of CS20, but emission patterns of pig slurry differed from cattle slurry. Less than 0.3% of the methane potential and only about 0.7% of the IPCC Tier 1 emission factor were emitted as CH_4_ during the first 3 days after excretion. In conclusion, quick removal of manure from the barn can reduce emissions, although immediate removal is not required from a CH_4_ emissions perspective.

## Plain Language Summary

When manure is stored, bacteria break down the organic matter and produce methane. Methane is a strong greenhouse gas that contributes to global warming. One way to reduce methane released from animal barns is to remove the manure more often. However, it has not been clear how fast the manure should be removed to minimize methane release. The goal of this research was to find out how much methane is released during the first 3 days after the manure is excreted by dairy cattle and pigs. Our results showed that methane emissions from cattle manure remained low and did not change during the first 3 days. In pig manure, methane emissions also started slow but increased over the 3 days. We also found that higher temperatures led to more methane being released. Although methane emissions are relatively low in the first days after excretion, our results support removing manure as soon as possible to reduce total methane emissions.

**Abbreviations**
BMP: biochemical methane potential
CRC: climate respiration chamber
CS15: cattle slurry with a room temperature of 15°C
CS20: cattle slurry with a room temperature of 20°C
DM: dry matter
PS20: pig slurry with a room temperature of 20°C
TN: total nitrogen
VS: volatile solids

## 1 INTRODUCTION

Methane (CH_4_) is a short‐lived greenhouse gas that has a major contribution to global warming. The global warming potential of non‐fossil CH_4_ for a time horizon of 100 years (GWP_100_) is currently estimated to be 27 times the GWP_100_ of carbon dioxide (CO_2_) (IPCC, 2021). In the European Union, over half of the CH_4_ emissions come from agriculture, including livestock (EEA, 2024). The major sources of CH_4_ emission in livestock production are enteric fermentation in ruminants and manure storage (Ward et al., 2024). About 20% of agricultural CH_4_ emissions originate from manure management (EEA, 2024).

During manure storage, CH_4_ is produced by anaerobic decomposition of organic matter. This process consists of four stages: hydrolysis, acidogenesis, acetogenesis, and methanogenesis. During the first three stages, complex organic matter is degraded and fermented into simple intermediates such as volatile fatty acids (VFAs) and alcohols, which are further converted into acetate, hydrogen, and CO_2_. In the final stage, methanogenic archaea produce CH_4_ through two main pathways: acetoclastic methanogens convert acetate into CH_4_ and CO_2_, while hydrogenotrophic methanogens reduce hydrogen and CO_2_ to CH_4_ (Zeeman, 1991). Although the majority of the methanogens have an enteric origin, the community in manure storage environments often differs from the original rumen community due to environmental selection (Habtewold et al., 2018; Ozbayram et al., 2018). Haeussermann et al. (2006) showed that when a manure pit was emptied and cleaned after a production round of fattening pigs, the next fattening period started with a low CH_4_ emission rate due to the complete lack of methane‐producing inoculum. This indicates that the methanogens need to adapt to the environmental differences between the gut and the manure storage, for example, temperature, ammonia level, and pH, before they produce CH_4_.

Studies have shown that reducing the time that slurry is stored inside the barn by frequently transferring manure to outside storages can reduce CH_4_ emissions from dairy and pig houses (Dalby et al., 2021; Hilgert et al., 2023). By quick removal of the excreted manure, less organic matter is degraded in the barn, and therefore less CH_4_ is produced in the barn. However, to prevent increased emissions from manure in outside storages, it is recommended to combine this removal strategy with anaerobic digestion or storage mitigation technologies (Dalby et al., 2021). Hilgert et al. (2023) showed that the biochemical methane potential (BMP) of fresh manure collected in the barn was higher compared to manure stored outside the barn for both pig and cattle manure. Thus, in addition to lower CH_4_ emissions from the barn and storage, anaerobic digestion of fresh manure can also increase biogas production.

This raises the question of how quickly the manure should be removed from the barn. Ammonia emissions are known to peak usually within 2 h after excretion (Elzing & Monteny, 1997). However, little is known about the breakdown of organic matter and the emission of CH_4_ in the first hours after excretion. Some studies measured CH_4_ emissions from manure storage tanks (Misselbrook et al., 2016; Wood et al., 2012), but they used manure that already had been stored before the start of the experiment. In contrast, Hilgert et al. (2022) used relatively fresh manure (max. 1 h old), but the emissions were not measured continuously, and the results do not show the development of CH_4_ emissions in the first hours after application of the manure. Therefore, it is not known how long it takes for the methanogens to adapt to the changing environment from gut to storage in the barn.

This study aimed to determine the breakdown of organic matter and emissions of CH_4_ from dairy cattle and pig slurry in the first 3 days after excretion. In Dutch practice, manure removal from barns ranges from hourly removal to intervals of up to several days. By collecting feces and urine right after excretion and using climate respiration chambers (CRCs), we were able to measure the amount of CH_4_ that was lost during the transition from gut fermentation to storage fermentation in the barn. This provides insight into how rapidly manure should be removed from the barn to maximize the biogas potential for anaerobic digestion.

## 2 MATERIALS AND METHODS

The experiment was conducted at the Carus research facility of Wageningen University and Research (Wageningen, The Netherlands) in September 2022. Gaseous emissions from dairy cattle and pig manure were measured in climate respiration chambers (CRCs). CRCs are the gold‐standard technique to determine the energy expenditure of individual animals (Tedeschi et al., 2022). They are generally used for research on energy metabolism (indirect calorimetry), nutrition, digestion, and gaseous emissions from animals. However, in the present study, only manure was added to the CRC, and no live animals were present.

### 2.1 Experimental setup in CRC

**FIGURE 1:**
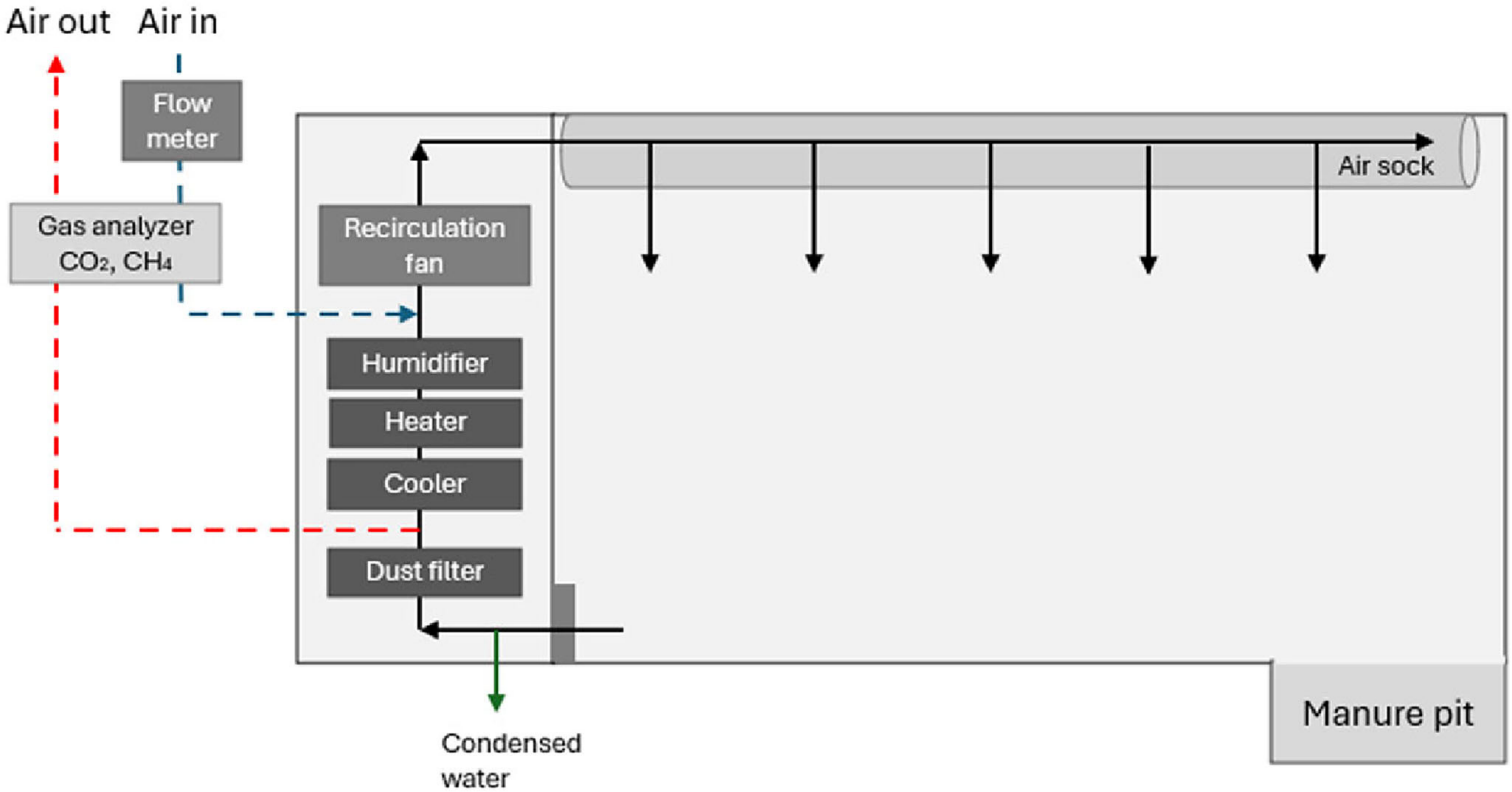
Schematic representation of the respiration chamber, based on Gaughan et al. (2015). Air is recirculated from the respiration chamber through the climate control unit (dust filter, cooler, heater, and humidifier) and then returned to the chamber. An air socket ensures uniform distribution of air entering the chamber. CO_2_ and CH_4_ concentrations in incoming and outgoing air were analyzed by a gas analyzer.

Three large CRCs were used, and each chamber was divided into two individual airtight compartments (11.8 m^2^, 35 m^3^). A detailed description of the CRC design and gas measurements has been reported by Heetkamp et al. (2015) and Gaughan et al. (2015). Briefly, gases were analyzed using a gas analyzer (ABB Advance Optima AO2000 systems, ABB) that measured CH_4_ and CO_2_ concentrations from the inlet and exhaust air from each compartment in 3 min intervals (Figure 1). The analyzer had a measurement range of 0–1000 ppm for CH_4_ with a detection limit of 1 ppm and 0%–1% for CO_2_ with a detection limit of 0.001%. Gas concentrations and ventilation rates were corrected for pressure, temperature, and relative humidity. CO_2_ and CH_4_ production in each CRC compartment was calculated from the difference between inlet and exhaust gas volumes. At the start of the experiment, each CRC compartment was checked by releasing known amounts of CO_2_ and comparing these values with the data from the gas analyses system to determine CO_2_ recovery. The average recovery of CO_2_ was 100.3% but ranged from 99.6% to 101.4% for individual compartments. This is well within the preferred range of 97%–103% (Danesh Mesgaran et al., 2020).

Each CRC compartment contained two manure pits with a square surface area of 1.3 m^2^ and depth of 0.5 m. During the experiment, only one manure pit was used. Prior to the start of the experiment, the manure pit was filled with old slurry that had previously been stored for several months (cattle slurry) or several weeks (pig slurry). After 4 days, most of the old manure was removed from the pit, leaving approximately 2 L remaining. This remaining manure functioned as an inoculum. In this way, a solid non‐slatted barn floor was simulated. Subsequently, fresh slurry (<1 h old) was added to the pit; this moment is referred to as *t* = 0. CH_4_ and CO_2_ emissions were measured for 72 h for dairy cattle slurry and 68 h for pig slurry.

Three treatments were studied: (1) dairy cattle slurry with a room temperature of 15°C (CS15), (2) dairy cattle slurry with a room temperature of 20°C (CS20), and (3) pig slurry with a room temperature of 20°C (PS20). The room temperatures were typical for animal housing in the Netherlands (Seedorf et al., 1998; Winkel et al., 2015). The higher temperature in the second treatment (CS20) was selected based on the assumption that a solid non‐slatted floor may lead to elevated temperatures on a solid floor compared to a slatted floor. In the first week, three CRC compartments were used for CS15 and three for CS20. In the second week, two compartments were used for PS20. The relative humidity was initially maintained at 65%. However, drying of the manure was observed at the edges of the manure pit, suggesting that the humidity level was insufficient. Consequently, the relative humidity was increased to 85% during the second week. The ventilation rate of each compartment was 14 m^3^/h. Air speed above the manure surface was measured at the start of the experiment and was about 0.2 m/s.

### 2.2 Slurry collection

Dairy cattle manure was collected from the experimental dairy farm of Carus research facility of Wageningen University and Research (Wageningen, the Netherlands). The Holstein Friesian cows were housed on a slatted floor with a manure pit below. Pig manure was collected from a commercial farrow‐to‐finish farm (De Heurne, The Netherlands). Feces and urine were separated on a manure belt with tiles below the slatted floor. The urine runs through the tiles and is stored separately from the feces.

The old dairy cattle slurry was collected from the manure pit after mixing. Old feces and urine from pigs were collected from the storages and were manually mixed into slurry with a urine:feces ratio of 4:3 (ter Elst‐Wahle & den Brok, 1995). Fresh feces and urine were collected upon excretion behind the animal for both dairy cattle and pigs and were directly cooled to 4°C to prevent emissions before the start of the experiment. Immediately before addition to the manure pit in the CRC, the fresh urine and feces were manually mixed into slurry with a urine:feces ratio of 5:8 for dairy slurry (Dai & Karring, 2014; de Boer, 2023) and 4:3 for pig slurry (ter Elst‐Wahle & den Brok, 1995). For practical reasons, the pig manure samples contained manure from both fattening pigs (75%) and farrowing sows (25%). The added manure volumes in the CRCs were based on the daily manure production per unit of manure pit surface in practice. This resulted in 16 L and 11.5 L slurry for dairy cattle and pigs, respectively, with a manure height of approximately 1.2 cm.

### 2.3 Manure samples and analyses

Manure samples were taken from the fresh slurry before it was applied to the manure pit of the CRC and at the end of the experiment. The samples were analyzed for dry matter (DM), ash, total nitrogen (TN), total ammoniacal nitrogen (TAN), total phosphorus (P), and pH in the laboratory of Wageningen Livestock Research (Wageningen, The Netherlands). Samples were dried at 105°C to a constant weight to determine DM, followed by dry combustion at 550°C to determine the ash content (NEN, 1998a). Volatile solid (VS) content was determined as the difference between DM and ash content. TN was analyzed by the Kjeldahl digestion method (NEN, 1998b), while TAN was analyzed by digestion and titration (NEN, 1998c). P content was analyzed by digestion and spectrophotometry (NEN, 2019) and was converted to P_2_O_5_ content by multiplying with 2.291. pH was measured with an electronic voltmeter (NEN, 2012).

From the fresh slurry, also the BMP was determined with an anaerobic batch test which was performed by LeAF BV (Wageningen, The Netherlands). Three samples of fresh cattle slurry and two samples of pig slurry were analyzed in duplicate. The BMP protocol followed in this study was in line with the principles and recommendations from Hafner et al. (2020). Briefly, 1 L vessels with 200 mL total liquid were used. The inoculum consisted of digestate from sewage sludge digestion. A control without substrate was executed to correct for endogenous biogas production from the inoculum. The BMP tests were conducted at 35°C, and the vessels were continuously stirred (100 rpm). Biogas production was monitored using a pressure‐based measurement system. BMP tests continued until the daily biogas production, corrected for gas production from the inoculum, was ≤0.5% of the cumulative biogas production (cattle slurry: 29 days, pig slurry: 44 days). Biogas composition was analyzed using a gas chromatograph at the end of the test. Additional gas composition analyses were performed when the internal pressure exceeded the operational limit and gas had to be released during the test (on day 7 for cattle slurry and on day 8 for pig slurry). The reported biogas volumes were corrected for dilution with the headspace gas. The pig slurry samples were inhomogeneous, resulting in large differences in biogas production during the test. Therefore, an additional BMP test was started 3 weeks after the initial tests, with gas composition measured on days 10 and 46.

### 2.4 Statistical analyses and calculations

Paired samples *t*‐tests were used to compare the manure composition from the same treatment at the start and end of the experiment. Differences in manure composition between cattle and pig slurry and cumulative emissions between the treatments were analyzed using independent samples *t*‐tests. To compare the cumulative emissions between CS15 and CS20, one‐tailed tests were applied based on the hypothesis that higher temperatures increase emissions. All other tests were two‐tailed. Homogeneity of variances was assessed using Levene's test, and Welch's adjustment was applied when necessary. Significance was set at *α* = 0.05, and all analyses were performed using IBM SPSS Statistics software.

The first‐order kinetic model is widely used to describe CH_4_ or biogas production in anaerobic digestion systems, assuming hydrolysis as the rate‐limiting step (Kafle & Chen, 2016; Roberts et al., 2023). The integrated form of the model, with time from 0 to *t* days and including the relationship between VS and CH_4_ production is (Kafle & Chen, 2016):

$${\mathrm{CH}}_{4}\;\left(t\right)={\mathrm{CH}}_{4}\_\max\times \left(1-e^{\left(-K_{h}t\right)}\right)$$

where CH_4_ (*t*) is cumulative CH_4_ yield at digestion time t (ml/g VS), CH_4__max is methane potential of the substrate (mL/g VS), hydrolysis constant (*K*_h_) is first order hydrolysis rate constant (day^−1^) and *t* is time (days). In this study, *K*_h_ was estimated by fitting the first‐order kinetic equation to the CH_4_ production data from the BMP analysis using the least squares method. The same method was applied to estimate the *K*_h_ from manure in the barn based on the cumulative CH_4_ emissions measured during the experiment.

## 3 RESULTS

### 3.1 Temperature

For CS15, the temperature in the CRC was set to 15°C, and the temperature of the manure in the manure pit was on average 15.2°C. For CS20 and PS20, the temperature in the CRC was set to 20°C; however, the temperature of the manure was on average only 19.0 and 18.8°C, respectively. Since the fresh manure was cooled prior to the experiment to prevent emissions, it required some time to adjust to the CRC temperature during the first hours of the experiment. Consequently, the manure temperature was slightly below the target of 15 or 20°C at the start. The first temperature measurements were taken after approximately 1 h, revealing minimum temperatures of, on average 14.6°C for CS15, 18.3°C for CS20, and 16.7°C for PS20. The manure temperature gradually reached the target temperatures, stabilizing after 4 h for CS15 and CS20 and 6 h for PS20. Since the temperature was already close to the target temperature within 1 h, these small deviations are expected to have little or no effect on the measured emissions.

### 3.2 Slurry characteristics

The slurry characteristics of CS15, CS20, and PS20 at the start and end of the experiment are presented in Table 1. DM and TAN concentration and the pH of PS20 were not significantly different from CS15 and CS20. In contrast, VS concentration in PS20 was significantly higher than in CS15 (*p* = 0.038) but was not different from CS20. Furthermore, TN and P_2_O_5_ concentrations were significantly higher in PS20 compared to CS15 (*p* = 0.003 and < 0.001, respectively) and CS20 (*p* = 0.005 and < 0.001, respectively).

**TABLE 1:** Slurry characteristics of the cattle slurry and pig slurry at the start and the end of the experiment (cattle slurry at atemperature of 15°C [CS15] was stored at 15°C and CS20 and pig slurry at a temperature of 20°C [PS20] were stored at 20°C). Dry matter (DM), volatile solids (VS), total nitrogen (TN), total ammoniacal nitrogen (TAN), and phosphate (P2O5) are expressed in g/kg. CS15 and CS20 were stored for 72 h and PS20 was stored for 68 h.

Slurry type							
Dairy cattle slurry		DM	VS	TN	TAN	P_2_O_5_	pH
CS15_start (*n* = 3)	Mean	108.1	89.2	4.0	0.5	1.5	7.4
	SD	3.2	4.6	0.2	<0.1	0.1	0.1
CS15_end (*n* = 3)	Mean	106.6	81.0	3.7	1.0	1.5	7.7
	SD	2.9	2.7	0.2	<0.1	0.1	0.3
CS20_start (*n* = 3)	Mean	106.6	88.2	4.2	0.7	1.6	7.3
	SD	1.4	0.9	0.3	0.1	0.1	0.1
CS20_end (*n* = 3)	Mean	101.9	77.4	3.5	0.9	1.5	7.4
	SD	4.6	2.6	0.1	0.1	0.1	0.2
**Pig slurry**							
PS20_start (*n* = 2)	Mean	127.1	105.3	6.0	2.4	4.0	7.7
	SD	6.0	5.7	0.3	0.3	0.2	0.4
PS20_end (*n* = 2)	Mean	112.8	90.7	4.5	1.6	3.8	7.1
	SD	1.1	0.9	0.2	0.2	0.1	0.1

The slurry characteristics changed during the experiment (Table 1). VS concentration decreased in both CS15 (*p* = 0.021) and CS20 (*p* = 0.023), but for PS20 this decrease was not significant. The decrease of VS content indicates the breakdown of organic matter. In CS15, CS20, and PS20, 9, 12, and 14 percent of the VS was degraded during the experiment, respectively; however, this was not significantly different between treatments. TN concentrations decreased in all three treatments, CS15 (*p* = 0.05), CS20 (*p* = 0.018), and PS20 (*p* = 0.008), while TAN only significantly increased in CS15 (*p* < 0.001) and decreased PS20 (*p* = 0.025). The rise in TAN demonstrates the mineralization of organic nitrogen to mineral nitrogen, and the loss of TN implies ammonia emissions. The phosphate concentration in CS15 and PS20 did not significantly change during the experiment, but in CS20 it significantly decreased (*p* = 0.020). Furthermore, the DM concentration and pH were not significantly different at the start compared to the end of the experiment.

### 3.3 Emissions

#### 3.3.1 Methane

**FIGURE 2:**
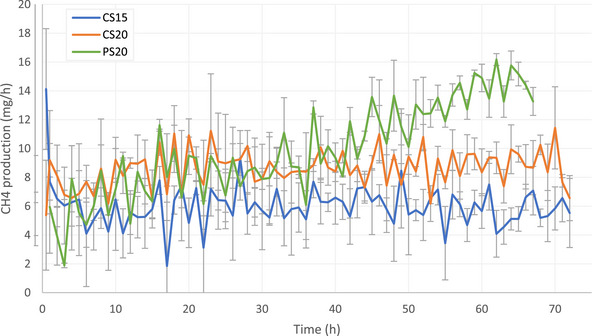
Average methane (CH_4_) production from cattle slurry at 15°C (blue), cattle slurry at 20°C (orange), and pig slurry at 20°C (green). Gray bars indicate standard deviation from the mean.

The CH_4_ production from dairy cattle and pig slurry showed a fluctuating trend (Figure 2). This occurred because the production of CH_4_ forms gas bubbles in the manure. When these bubbles come into contact with the manure surface, they pop, releasing CH_4_ into the atmosphere (Kupper et al., 2020). The amount of CH_4_ measured in the CRC depends on the number of gas bubbles that popped during a measuring period, and this created a fluctuating trend in CH_4_ production.

Despite the fluctuations, the average CH_4_ emissions from CS15 and CS20 remained constant in the first 72 h (Figure 1), although the emission rate from CS20 was higher than it was from CS15 (8.6 and 6.0 mg/h, respectively). The CH_4_ emissions from PS20 gradually increased from about 4 to 15 mg/h in the first 68 h.

**FIGURE 3:**
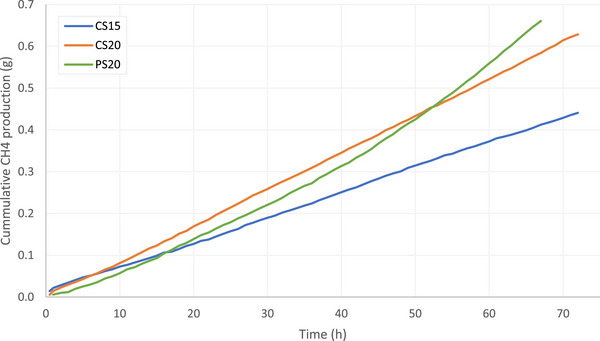
Cumulative methane (CH_4_) production from cattle slurry at 15°C (blue), cattle slurry at 20°C (orange), and pig slurry at 20°C (green).

The cumulative CH_4_ emissions from CS15 and CS20 showed a linear trend, while the cumulative CH_4_ emission from PS20 showed an exponential trend in the first 3 days after excretion (Figure 3). After 68 h, 0.41 g CH_4_ emitted from CS15, 0.58 g from CS20, and 0.66 g from PS20. From 8 h after excretion, the cumulative CH_4_ emissions from CS20 were higher compared to CS15, and this difference increased over time. Cumulative CH_4_ emissions from CS20 were significantly higher than those from CS15 after 39 h (*p* = 0.041). Even though the cumulative CH_4_ emission from PS20 showed an exponential increase and was higher compared to that from CS20 after 68 h, this difference was not significant from CS20. In contrast, the CH_4_ emission from PS20 was significantly higher than from CS15 after 60 h (*p* = 0.039). Notably, the cumulative CH_4_ emission from PS20 was significantly lower than that from CS20 between 2 and 8 h after excretion (*p*‐values ranging from 0.008 to 0.045).

The calculated *K*_h_ for fresh manure on a solid non‐slatted floor in the first 68 h after excretion was on average 0.004, 0.005, and 0.004 day^−1^ and the associated maximum CH_4_ production was on average 71, 65, and 117 m^3^/tonne VS for CS15, CS20, and PS20, respectively.

#### 3.3.2 Carbon dioxide

**FIGURE 4:**
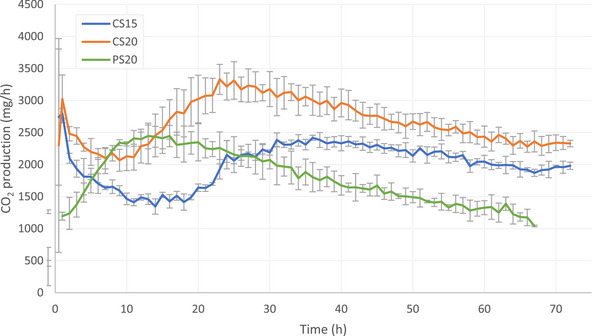
Average carbon dioxide (CO_2_) production from cattle slurry at 15°C (blue), cattle slurry at 20°C (orange), and pig slurry at 20°C (green). Gray bars indicate standard deviation from the mean.

The CO_2_ production from CS15 and CS20 decreased in the first 10 to 15 h after application of the fresh slurry (Figure 4). Again, CO_2_ production from CS20 was higher compared to that from CS15. The CO_2_ production from CS20 peaked after 25 h at a rate of 3313 mg/h, while the CO_2_ production from CS15 peaked after 37 h at a rate of 2383 mg/h. Then, CO_2_ production decreased during the remainder of the measuring period. The CO_2_ production from PS20 increased to 2245 mg/h in the first 12 h after application of the fresh slurry, after which a linear decrease persisted during the rest of the measuring period. After 68 h, CO_2_ production was 1914, 2291, and 1041 mg/h for CS15, CS20, and PS20, respectively.

**FIGURE 5:**
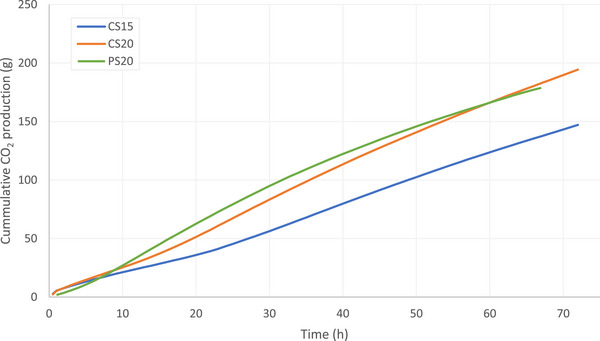
Cumulative carbon dioxide (CO_2_) production from cattle slurry at 15°C (blue), cattle slurry at 20°C (orange), and pig slurry at 20°C (green).

The cumulative CO_2_ emission from CS15 was lower compared to that from CS20 and PS20 (Figure 5). The trends are not fully linear due to the changing emission rates over time (Figure 3). From 7 h after excretion until the end of the experiment, the CO_2_ emission from CS20 was significantly higher than from CS15 (*p*‐values ranging from < 0.001 to 0.036). From 12 to 25 and 34 h after excretion, PS20 showed significantly higher cumulative CO_2_ emissions compared to CS20 and CS15, respectively. However, the cumulative CO_2_ emissions from CS20 exceed those from PS20, due to the decrease in emission rate from PS20 after 60 h, but this difference was not significant. After 68 h, 137, 183, and 179 g CO_2_ emitted from CS15, CS20, and PS20, respectively.

#### 3.3.3 Proportion of CH_4_‐C to total lost carbon

**FIGURE 6:**
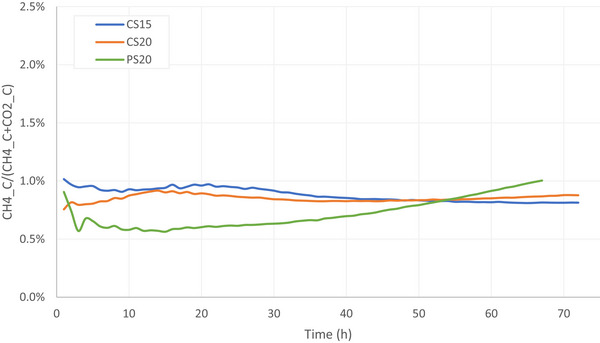
Proportion of CH_4_‐C in total C lost (CH_4_‐C+CO_2_‐C) based on cumulative emissions per hour in cattle slurry at 15°C (blue), cattle slurry at 20°C (orange), and pig slurry at 20°C (green).

In the first 68 h, CH_4_‐C accounted for <1.0% of the total carbon losses (CH_4_‐C + CO_2_‐C) in both cattle slurry at both temperatures, as well as in pig slurry (Figure 6). CS15 showed the highest initial CH_4_‐C proportion at the start, but this appeared to decrease toward the end of the experiment. In CS20, the CH_4_‐C proportion slightly increased to 0.92% at 14 h after application of fresh slurry and then gradually decreased, stabilizing after approximately 35 h. No significant differences were observed between the CH_4_‐C proportion of CS15 and CS20 at any point during the experiment.

From PS20, the CH_4_‐C proportion developed differently from that of cattle slurry. During the first few hours, the ratio decreased to a minimum of 0.56%, but after approximately 20 h, it began to increase, and this upward trend continued until the end of the experiment. This suggests that, after roughly 1 day, relatively more carbon is lost as CH_4_ compared to the initial 24 h. After 54 h, the CH_4_‐C proportion was similar across all three treatments (0.84%). Beyond this point, the proportion from CS20 remained constant, that from PS20 continued to increase, and that from CS15 continued to decrease. From 14 to 36 h after application of the fresh slurry, the CH_4_‐C proportion from PS20 was significantly lower than that from CS20 (*p*‐values ranging from 0.019 to 0.047).

#### 3.3.4 Biochemical methane potential

The maximum biogas production from cattle and pig slurry was on average 387 and 449 Nm^3^/tonne VS, respectively (Table 2). The biogas from cattle slurry contained 64% CH_4_, while the biogas from pig slurry contained 62% CH_4_. Based on the maximum biogas production, the calculated *K*_h_ of fresh cattle and pig slurry during digestion were on average 0.15 and 0.12, respectively.

**TABLE 2:** Measured biochemical methane potential (BMP) and calculated hydrolysis constant (*K*_h_) in day^−1^ and theoretical maximum methane production (CH_4__max) in m^3^ biogas/tonne volatile solids (VS) of cattle and pig slurry.

	Biogas production					Calculated	
Sample	Nm^3^/tonne VS		Nm^3^/tonne substrate		% CH_4_ in biogas	*K*_h_	CH_4__max
Cattle slurry	382	±1.1	34	±0.1	64	0.15	432
Cattle slurry	409	±0.5	40	±0.1	64	0.16	454
Cattle slurry	369	±3.5	36	±0.3	64	0.13	430
Pig slurry	412	±45	39	±4.3	62	0.13	404
Pig slurry	485	±39	48	±3.1	62	0.11	480

## 4 DISCUSSION

### 4.1 Slurry characteristics

For dairy cattle manure, the DM and VS concentration of the fresh manure at the start of the experiment was higher compared to relatively fresh manure in the literature (Cárdenas et al., 2021; Hilgert et al., 2023; Petersen et al., 2012). TN concentration was comparable with the literature (Petersen et al., 2012), although TAN concentration was lower (Hilgert et al., 2023; Petersen et al., 2012) and even less than TAN in manure stored in tanks or barns (Kupper et al., 2020; Prado et al., 2022). Dai and Karring (2014) showed that the TAN concentration in pig and cattle slurry rapidly increased in the first few hours after mixing feces and urine and stabilized after about 30 h. This increase indicates that the TAN in the fresh manure of the present research was not yet formed, and this also explains the increased TAN concentration in the manure at the end of the experiment. The pH of the starting manure was in line with reported pH values from commercial farms (Prado et al., 2022) but was higher compared to the pH of the fresh manure used by Hilgert et al. (2023). For pig slurry, the DM concentration was comparable with the literature, while VS concentration was much higher (Hilgert et al., 2023). This difference may partly be explained by the assumed urine‐to‐feces ratio of 4:3 in the present study, whereas in other studies a 3:1 ratio is adopted (Dai & Karring, 2014; Dalby et al., 2023). A higher fecal fraction not only increases the VS concentration but also provides more degradable organic matter for methanogenesis, which may result in higher CH_4_ production potential compared to practical conditions. TN concentration in pig slurry was comparable with values from commercial farms (Prado et al., 2022). Reported TAN concentration in manure in the literature is not consistent and ranges from 1.5 to 3.7 g/kg (Hilgert et al., 2023; Kupper et al., 2020; Prado et al., 2022). Hence, in this research the TAN concentration of the pig manure fell within this range. However, pH was slightly higher than in the literature (Hilgert et al., 2023).

### 4.2 Methane emissions

The results showed that the emission rate from CS20 was higher than from CS15. The effect of temperature on emissions has been reported in several studies. For example, Im et al. (2020) found increased CH_4_ missions from manure with higher temperatures in an incubation experiment with temperatures ranging from 15 to 35°C. Also, Dalby et al. (2021) concluded that slurry temperature is one of the most important factors controlling CH_4_ production in manure management systems. Elsgaard et al. (2016) derived the temperature dependency of CH_4_ production from cattle and pig slurry with the Arrhenius equation. They showed that when the temperature increased by 10°C in the range between 5 and 37°C, the CH_4_ production rate increased by a factor of 3.4 (Q10). In the present study, the CH_4_ production rate increased with a factor of 1.4 when the temperature was about 4°C higher, which was comparable with the Q10 found by Elsgaard et al. (2016).

The observed CH_4_ production from cattle and pig slurry during the experimental period reached only a limited fraction of the BMP of the slurries. The CH_4_ lost in the first 68 h after excretion compared to the BMP was only 0.18%, 0.25%, and 0.29% for CS15, CS20, and PS20, respectively. When compared to the IPCC Tier 1 emission factors for liquid/slurry storage in warm temperate climates (IPCC, 2019), the CH_4_ emissions from CS15, CS20, and PS20 were only 0.5%, 0.7%, and 0.5% of the annual emission per kg of organic matter, respectively. This indicates that only a small portion of the methane potential was realized, showing that quick removal of manure from the barn can largely prevent CH_4_ emissions from the barn while preserving most of the methane potential for subsequent biogas production during anaerobic digestion.

### 4.3 Proportion of CH_4_‐C to total lost carbon

In this study, the proportion of CH_4_ relative to the total carbon loss (CH_4_‐C + CO_2_‐C) was less than 1%. This is considerably lower than the proportion of CH_4_ found in the biogas produced during the BMP analyses. Emission ratios of CH_4_ and CO_2_ from manure storage are known to be highly variable (Petersen, 2018). However, in relatively fresh manure, the CH_4_/CO_2_ ratio can be low due to the absence of an established methanogenic community (Dalby et al., 2021). Sommer et al. (2007) reported CH_4_‐C/(CH_4_‐C + CO_2_‐C) ratios of 10%–30% in slurry made from freshly collected urine and feces (1–2 h old) from cattle and pigs at 10°C. These ratios increased to 20%–50% in cattle slurry and up to 65% in pig slurry at 20°C. The low proportion of CH_4_ observed in the present study may be explained by the absence of anaerobic conditions on a solid non‐slatted floor, which restricts CH_4_ formation.

The proportion of CH_4_ relative to the total carbon from CS15 and CS20 loss remained constant during the first 68 h after excretion. In contrast, the CH_4_ proportion from PS20 began to increase rapidly after approximately 24 h. This suggests that the methanogenic community might establish more quickly in pig slurry than in cattle slurry.

In the above calculation of the CH_4_‐C to total carbon lost, total carbon losses were assumed to equal the sum of CH_4_‐C and CO_2_‐C. However, this assumption likely underestimates total carbon losses, as additional pathways, such as emissions of other volatile organic compounds, including VFAs, were not captured in the gas measurements. Emission fluxes and the types of volatile organic compounds from manure are highly variable and not yet fully understood (Ni et al., 2012), indicating that carbon losses from manure are not limited to CH_4_ and CO_2_.

### 4.4 Biochemical methane potential

The maximum biogas production from fresh dairy cattle in the present study was on average 387 m^3^/tonne VS (248 m^3^ CH_4_/tonne VS). This is higher than the average BMP of 220 m^3^ CH_4_/tonne VS reported by Groenestein et al. (2016), who reviewed several studies that analyzed the BMP from cattle and pig manure. Hilgert et al. (2023) found higher BMPs from cattle manure sampled directly from the barn, ranging from about 225 to 325 m^3^/tonne VS. The BMPs analyzed in the present study fall within this range. The average biogas production from pig slurry in the present study was 449 m^3^/tonne VS (278 m^3^ CH_4_/tonne VS). This is lower than the average BMP for pigs reported by Groenestein et al. (2016) and Timmerman et al. (2009), which was 310 and 300 m^3^ CH_4_/tonne VS, but within the range of 260–405 m^3^/tonne VS from the study of Hilgert et al. (2023). This confirms that the anaerobic digestion of fresh cattle manure results in more biogas production compared to the digestion of manure that has previously been stored; however, this trend is less clear for pig slurry.

The hydrolysis rate constant (*K*_h_) is used in models to calculate how much organic matter is converted into CH_4_ within a certain time span. In the present study, the *K*_h_ during digestion was derived from the cumulative CH_4_ production during the BMP analysis. Timmerman et al. (2009) used the same method and found values of *K*_h_ ranging from 0.07 to 0.27 day^−1^ with maximum CH_4_ production ranging from 213 to 596 m^3^ CH_4_/tonne VS for different types of pig manure and treatments. In the model from Gollenbeek et al. (2021), a *K*_h_ of 0.156 day^−1^ was used for pig slurry during anaerobic digestion. For CH_4_ production in the barn, a *K*_h_ of 0.009 day^−1^ was used, based on the assumption that 62.2% of the BMP is released in the barn. In a comparable study for dairy cattle, Gollenbeek et al. (2022) also assumed that the *K*_h_ for manure in cattle housing is comparable with that for in pig housing, since there was no specific *K*_h_ for dairy cattle housing. In the present study, the *K*_h_ during anaerobic digestion of pig slurry and the *K*_h_ from the barn of both pig and cattle slurry were lower compared to the *K*_h_ values used by Gollenbeek et al. (2021, 2022). The values of *K*_h_ reported in the present study were calculated using only CH_4_ and CO_2_ production, excluding intermediate VFAs. As a result, these *K*_h_ values likely underestimate the hydrolysis rate, which explains why they are somewhat lower than those reported in literature. Still, it is reasonable that the *K*_h_ in the present study is lower compared to literature, as it is based on emissions from only the first 3 days after excretion, and our experimental setup was based on a different housing system. Therefore, to model emissions from innovative housing systems with frequent removal of manure, it would be more accurate to use a lower *K*_h_.

In conclusion, the breakdown of organic matter and emissions of CH_4_ from both dairy cattle and pig slurry were minimal in the first 3 days after excretion. Less than 0.3% of the methane potential was emitted as CH_4_ and CH_4__C accounted for <1.0% of the total carbon losses (CH_4_‐C + CO_2_‐C). Both CH_4_ and CO_2_ emissions from CS20 were higher than from CS15; therefore, a higher temperature leads to increasing emission rates. Although cumulative CH_4_ and CO_2_ emissions from PS20 were not significantly different from those of CS20, pig slurry showed different emission patterns. CH_4_ emissions started increasing immediately after excretion, and the proportion of CH_4_ relative to CO_2_ began to increase after 24 h. This suggests that the adaptation from gut fermentation to storage fermentation is progressing in the first 3 days after excretion.

Since emissions start immediately after excretion, barn emissions can be reduced by removing manure as quickly as possible. However, from a CH_4_ emission perspective, it is not necessary to remove the manure within at least 3 days after excretion. It should nevertheless be emphasized that ammonia emissions can occur at a faster rate, which requires quicker manure removal from the barn to reduce the overall environmental impact.

## AUTHOR CONTRIBUTIONS

**E. G. G. van Boxmeer**: Conceptualization; data curation; formal analysis; methodology; writing—original draft; writing—review and editing. **H. J. Smit**: Conceptualization; writing—original draft; writing—review and editing. **N. Verdoes**: Conceptualization; funding acquisition; methodology; project administration; supervision; writing—review and editing.

## CONFLICT OF INTEREST STATEMENT

The authors declare no conflicts of interest.

## References

[jeq270186-bib-0001] Cárdenas, A. , Ammon, C. , Schumacher, B. , Stinner, W. , Herrmann, C. , Schneider, M. , Weinrich, S. , Fischer, P. , Amon, T. , & Amon, B. (2021). Methane emissions from the storage of liquid dairy manure: Influences of season, temperature and storage duration. Waste Management, 121, 393–402. 10.1016/j.wasman.2020.12.026 33445112

[jeq270186-bib-0002] Dai, X. , & Karring, H. (2014). A determination and comparison of urease activity in feces and fresh manure from pig and cattle in relation to ammonia production and pH changes. PLoS One, 9(11), e110402. 10.1371/journal.pone.0110402 25397404 PMC4232307

[jeq270186-bib-0003] Dalby, F. R. , Ambrose, H. W. , Poulsen, J. S. , Nielsen, J. L. , & Adamsen, A. P. S. (2023). Pig slurry organic matter transformation and methanogenesis at ambient storage temperatures. Journal of Environmental Quality, 52(6), 1139–1151. 10.1002/jeq2.20512 37703095

[jeq270186-bib-0004] Dalby, F. R. , Hafner, S. D. , Petersen, S. O. , VanderZaag, A. C. , Habtewold, J. , Dunfield, K. , Chantigny, M. H. , & Sommer, S. G. (2021). Understanding methane emission from stored animal manure: A review to guide model development. Journal of Environmental Quality, 50(4), 817–835. 10.1002/jeq2.20252 34021608

[jeq270186-bib-0005] Danesh Mesgaran, S. , Frydendahl Hellwing, A. L. , Lund, P. , Derno, M. , Kuhla, B. , Heetkamp, M. , Miller, G. , Humphries, D. , Anglard, F. , Rochette, Y. , Martin, C. , Gardiner, T. , & Coleman, M. (2020). The gas recovery test of respiratory chambers. In S. D. Mesgaran , R. Baumont , L. Munksgaard , D. Humphries , E. Kennedy , J. Dijkstra , R. Dewhurst , H. Ferguson , M. Terré , & B. Kuhla (Eds.), Methods in cattle physiology and behaviour—Recommendations from the SmartCow consortium. PUBLISSO. 10.5680/mcpb010

[jeq270186-bib-0006] de Boer, H. (2023). Niveau en samenstelling van het stikstofverlies uit een melkveestal met roostervloer en een koetoilet . 10.18174/640152

[jeq270186-bib-0007] EEA . (2024). Annual European Union greenhouse gas inventory 1990 – 2022 and inventory document 2024; First submission under the Enhanced Transparency Framework of the Paris Agreement . https://www.eea.europa.eu/

[jeq270186-bib-0008] Elsgaard, L. , Olsen, A. B. , & Petersen, S. O. (2016). Temperature response of methane production in liquid manures and co‐digestates. Science of the Total Environment, 539, 78–84. 10.1016/j.scitotenv.2015.07.145 26356180

[jeq270186-bib-0009] Elzing, A. , & Monteny, G. J. (1997). Ammonia emission in a scale model of a dairy‐cow house. Transactions of the American Society of Agricultural Engineers, 40(3), 713–720. 10.13031/2013.21301

[jeq270186-bib-0010] Gaughan, J. B. , Heetkamp, M. J. W. , & Hendriks, P. (2015). Indirect calorimetry: Assessing animal response to heat and cold stress. Indirect calorimetry. Brill | Wageningen Academic.

[jeq270186-bib-0011] Gollenbeek, L. , van Gastel, J. , Casu, F. , Huisman, I. , & Verdoes, N. (2022). Berekeningen emissies en economie voor verschillende scenario's voor verwaarding van rundveemest (NL next level Mestverwaarden). Wageningen University & Research.

[jeq270186-bib-0012] Gollenbeek, L. , van Gastel, J. , Casu, F. , & Verdoes, N. (2021). Emissies en kosten van verschillende scenario's voor verwaarding van varkensmest (NL next level Mestverwaarden). Wageningen University & Research.

[jeq270186-bib-0013] Groenestein, C. M. , Mosquera, J. , & Melse, R. W. (2016). Methaanemissie uit mest : schatters voor biochemisch methaan potentieel (BMP) en methaanconversiefactor (MCF) . 10.18174/401705

[jeq270186-bib-0014] Habtewold, J. , Gordon, R. , Sokolov, V. , VanderZaag, A. , Wagner‐Riddle, C. , & Dunfield, K. (2018). Targeting bacteria and methanogens to understand the role of residual slurry as an inoculant in stored liquid dairy manure. Applied and Environmental Microbiology, 84(7), 17. 10.1128/AEM.02830-17 PMC5861833 29374043

[jeq270186-bib-0015] Haeussermann, A. , Hartung, E. , Gallmann, E. , & Jungbluth, T. (2006). Influence of season, ventilation strategy, and slurry removal on methane emissions from pig houses. Agriculture, Ecosystems & Environment, 112, 115–121. 10.1016/j.agee.2005.08.011

[jeq270186-bib-0016] Hafner, S. D. , de Laclos, H. F. , Koch, K. , & Holliger, C. (2020). Improving inter‐laboratory reproducibility in measurement of biochemical methane potential (BMP). Water, 12(6), 1752. 10.3390/w12061752

[jeq270186-bib-0017] Heetkamp, M. J. W. , Alferink, S. J. J. , Zandstra, T. , Hendriks, P. , van den Brand, H. , & Gerrits, W. J. J. (2015). Design of climate respiration chambers, adjustable to the metabolic mass of subjects. In Indirect calorimetry (pp. 35–56) Brill | Wageningen Academic.

[jeq270186-bib-0018] Hilgert, J. E. , Amon, B. , Amon, T. , Belik, V. , Dragoni, F. , Ammon, C. , Cárdenas, A. , Petersen, S. O. , & Herrmann, C. (2022). Methane emissions from livestock slurry: Effects of storage temperature and changes in chemical composition. Sustainability, 14(16), 9934. 10.3390/su14169934

[jeq270186-bib-0019] Hilgert, J. E. , Herrmann, C. , Petersen, S. O. , Dragoni, F. , Amon, T. , Belik, V. , Ammon, C. , & Amon, B. (2023). Assessment of the biochemical methane potential of in‐house and outdoor stored pig and dairy cow manure by evaluating chemical composition and storage conditions. Waste Management, 168, 14–24. 10.1016/j.wasman.2023.05.031 37276630 PMC10470457

[jeq270186-bib-0020] Im, S. , Petersen, S. O. , Lee, D. , & Kim, D. H. (2020). Effects of storage temperature on CH_4_ emissions from cattle manure and subsequent biogas production potential. Waste Management, 101, 35–43. 10.1016/j.wasman.2019.09.036 31586875

[jeq270186-bib-0021] IPCC . (2019). Emissions from livestock and manure management. In *2019 Refinement to the 2006 IPCC Guidelines for National Greenhouse Gas Inventories, volume 4 (agriculture, forestry and other land use)*. IPCC.

[jeq270186-bib-0022] IPCC . (2021). Climate change 2021: The physical science basis. In P. A. Arias , N. Bellouin , E. Coppola , R. G. Jones , G. Krinner , J. Marotzke , V. Naik , M. D. Palmer , G.‐K. Plattner , J. Rogelj , M. Rojas , J. Sillmann , T. Storelvmo , P. W. Thorne , B. Trewin , K. Achuta Rao , B. Adhikary , R. P. Allan , K. Armour , … B. Zhou (Eds.), Contribution of Working Group I to the Sixth Assessment Report of the Intergovernmental Panel on Climate Change (pp. 33–144). Cambridge University Press. 10.1017/9781009157896.002

[jeq270186-bib-0023] Kafle, G. K. , & Chen, L. (2016). Comparison on batch anaerobic digestion of five different livestock manures and prediction of biochemical methane potential (BMP) using different statistical models. Waste Management, 48, 492–502. 10.1016/j.wasman.2015.10.021 26531046

[jeq270186-bib-0024] Kupper, T. , Häni, C. , Neftel, A. , Kincaid, C. , Bühler, M. , Amon, B. , & VanderZaag, A. (2020). Ammonia and greenhouse gas emissions from slurry storage—A review. Agriculture, Ecosystems & Environment, 300, 106963. 10.1016/j.agee.2020.106963

[jeq270186-bib-0025] Misselbrook, T. , Hunt, J. , Perazzolo, F. , & Provolo, G. (2016). Greenhouse gas and ammonia emissions from slurry storage: Impacts of temperature and potential mitigation through covering (pig slurry) or acidification (cattle slurry). Journal of Environmental Quality, 45(5), 1520–1530. 10.2134/jeq2015.12.0618 27695736

[jeq270186-bib-0026] NEN . (1998a). NEN 7432: Manure and derivatives—Determination of the contents of dry matter and organic matter—Gravimetric method . Nederlands Normalisatie‐instituut. https://www.nen.nl/nen-7432-1998-nl-31721

[jeq270186-bib-0027] NEN . (1998b). NEN 7434: Manure and derivatives—Determination of the nitrogen content in digests. Nederlands Normalisatie‐instituut . https://www.nen.nl/nen-7434-1998-nl-31723

[jeq270186-bib-0028] NEN . (1998c). NEN 7438: Manure and derivatives—Determination of the ammoniacal nitrogen content—Titrimetric method . Nederlands Normalisatie‐instituut. https://www.nen.nl/nen-7438-1998-nl-35428

[jeq270186-bib-0029] NEN . (2012). NEN‐EN‐ISO 10523: Water quality—Determination of pH . Nederlands Normalisatie‐instituut. https://www.nen.nl/nen-en-iso-10523-2012-en-168549

[jeq270186-bib-0030] NEN . (2019). NEN 7435: Manure and derivatives—Determination of the phosphorus content in digests . Nederlands Normalisatie‐instituut. https://www.nen.nl/nen-7435-2019-nl-265336

[jeq270186-bib-0031] Ni, J.‐Q. , Robarge, W. P. , Xiao, C. , & Heber, A. J. (2012). Volatile organic compounds at swine facilities: A critical review. Chemosphere, 89(7), 769–788. 10.1016/j.chemosphere.2012.04.061 22682363

[jeq270186-bib-0032] Ozbayram, E. , Ince, O. , Ince, B. , Harms, H. , & Kleinsteuber, S. (2018). Comparison of rumen and manure microbiomes and implications for the inoculation of anaerobic digesters. Microorganisms, 6(1), 15. 10.3390/microorganisms6010015 29443879 PMC5874629

[jeq270186-bib-0033] Petersen, S. O. (2018). Greenhouse gas emissions from liquid dairy manure: Prediction and mitigation. Journal of Dairy Science, 101(7), 6642–6654. 10.3168/jds.2017-13301 29224872

[jeq270186-bib-0034] Petersen, S. O. , Andersen, A. J. , & Eriksen, J. (2012). Effects of cattle slurry acidification on ammonia and methane evolution during storage. Journal of Environmental Quality, 41(1), 88–94. 10.2134/jeq2011.0184 22218177

[jeq270186-bib-0035] Prado, J. , Ribeiro, H. , Alvarenga, P. , & Fangueiro, D. (2022). A step towards the production of manure‐based fertilizers: Disclosing the effects of animal species and slurry treatment on their nutrients content and availability. Journal of Cleaner Production, 337, 130369. 10.1016/j.jclepro.2022.130369

[jeq270186-bib-0036] Roberts, S. , Mathaka, N. , Zeleke, M. A. , & Nwaigwe, K. N. (2023). Comparative analysis of five kinetic models for prediction of methane yield. Journal of the Institution of Engineers (India): Series A, 104(2), 335–342. 10.1007/s40030-023-00715-y

[jeq270186-bib-0037] Seedorf, J. , Hartung, J. , Schröder, M. , Linkert, K. H. , Pedersen, S. , Takai, H. , Johnsen, J. O. , Metz, J. H. M. , Groot Koerkamp, P. W. G. , Uenk, G. H. , Phillips, V. R. , Holden, M. R. , Sneath, R. W. , Short, J. L. , White, R. P. , & Wathes, C. M. (1998). Temperature and moisture conditions in livestock buildings in Northern Europe. Journal of Agricultural Engineering Research, 70(1), 49–57. 10.1006/jaer.1997.0284

[jeq270186-bib-0038] Sommer, S. G. , Petersen, S. O. , Sørensen, P. , Poulsen, H. D. , & Møller, H. B. (2007). Methane and carbon dioxide emissions and nitrogen turnover during liquid manure storage. Nutrient Cycling in Agroecosystems, 78(1), 27–36. 10.1007/s10705-006-9072-4

[jeq270186-bib-0039] Tedeschi, L. O. , Abdalla, A. L. , Álvarez, C. , Anuga, S. W. , Arango, J. , Beauchemin, K. A. , Becquet, P. , Berndt, A. , Burns, R. , De Camillis, C. , Chará, J. , Echazarreta, J. M. , Hassouna, M. , Kenny, D. , Mathot, M. , Mauricio, R. M. , McClelland, S. C. , Niu, M. , Onyango, A. A. , … Kebreab, E. (2022). Quantification of methane emitted by ruminants: A review of methods. Journal of Animal Science, 100(7), skac197. 10.1093/jas/skac197 35657151 PMC9261501

[jeq270186-bib-0040] ter Elst‐Wahle, E. R. , & den Brok, G. M. (1995). Gescheiden afvoer van urine en feces in combinatie met spoelen bij vleesvarkens . https://edepot.wur.nl/48936

[jeq270186-bib-0041] Timmerman, M. , van Riel, J. , Bisschops, I. , & van Eekert, M. (2009). Optimaliseren van mestvergisting .

[jeq270186-bib-0042] Ward, N. , Atkins, A. , & Atkins, P. (2024). Estimating methane emissions from manure: A suitable case for treatment? Environmental Research: Food Systems, 1(2), 025003. 10.1088/2976-601x/ad64d7

[jeq270186-bib-0043] Winkel, A. , Mosquera, J. , Groot Koerkamp, P. W. G. , Ogink, N. W. M. , & Aarnink, A. J. A. (2015). Emissions of particulate matter from animal houses in the Netherlands. Atmospheric Environment, 111, 202–212. 10.1016/j.atmosenv.2015.03.047

[jeq270186-bib-0044] Wood, J. D. , Gordon, R. J. , Wagner‐Riddle, C. , Dunfield, K. E. , & Madani, A. (2012). Relationships between dairy slurry total solids, gas emissions, and surface crusts. Journal of Environmental Quality, 41(3), 694–704. 10.2134/jeq2011.0333 22565251

[jeq270186-bib-0045] Zeeman, G. (1991). Mesophilic and psychrophilic digestion of liquid manure . 10.18174/202851

